# Diphtheria antitoxin treatment: from pioneer to neglected

**DOI:** 10.1590/0074-02760240214

**Published:** 2025-01-20

**Authors:** Lucia Grandière Pérez, Sylvain Brisse

**Affiliations:** 1 Service des Maladies Infectieuses et Tropicales, Centre Hospitalier Le Mans, Le Mans, France; 2 Université d’Angers, Angers, France; 3 Institut Pasteur, Université Paris Cité, Biodiversity and Epidemiology of Bacterial Pathogens, Paris, France; 4 Institut Pasteur, French National Reference Centre for Corynebacteria of the Diphtheriae complex, Paris, France

**Keywords:** Corynebacterium diphtheriae, Corynebacterium ulcerans, diphtheria toxin, taxonomy, clinical guidelines, epidemiology

## Abstract

Diphtheria, a severe respiratory infection, was a major killer of children until the early years of the 20th century. Although diphtheria is now largely controlled globally thanks to vaccination, it is still endemic in some world regions and large epidemics can occur where vaccination coverage is insufficient. The pathological effects caused by its main virulence factor, diphtheria toxin, can be diminished by passive transfer of antibodies. Equine diphtheria antitoxin (eDAT), the cornerstone of treatment against toxinic complications of diphtheria, was invented more than 130 years ago, in 1890, and is still in use today. A method to concentrate anti-diphtheria antibodies from hyperimmune equine serum was described in the first issue of Memórias do Instituto Oswaldo Cruz in 1909. On this historic occasion, we present recent knowledge on taxonomic, epidemiological and clinical aspects of diphtheria agents that produce diphtheria toxin, and provide a historical perspective on eDAT treatment, adverse effects, threats on its scarce international supply, and current avenues for alternative therapeutic strategies.

The burden of diphtheria has been largely reduced thanks to large-scale vaccination against diphtheria toxin (DT), antimicrobial therapy, and a therapeutic product specific to this disease, known as diphtheria antitoxin (DAT), or more specifically equine DAT (eDAT) when obtained from horses. eDAT was the first discovered curative treatment for diphtheria, described by von Behring in 1890 and inspired by his work in common with Kitasato on a way to neutralise tetanus toxin. The first Nobel Prize for Medicine was awarded to Emil von Behring in 1901 for this breakthrough.^1^ ^,^ ^2^ Today, eDAT still remains critical to reduce morbidity and mortality caused by the effect of diphtheria toxin on the human body.^3^ But eDAT, being produced from horse serum, has frequent adverse effects, including anaphylactic reactions to horse proteins, fever and serum sickness. Attempts to purify and concentrate anti-DT antibodies from eDAT were therefore deployed. In 1909, in the first edition of the journal “Memórias do Instituto Oswaldo Cruz”, Giemsa and Godoy described a method of ultrafiltration of horse serum based on the use of agar membranes.^4^ This method allowed the concentration of antibodies against diphtheria toxin. Despite the early recognition of the issue of adverse effects of eDAT safety for patients, equine serum production still poses challenges nowadays.^5^ ^,^ ^6^

Here, we provide updates on diphtheria microbiology, epidemiology and clinical aspects, and present the history of the role of eDAT in diphtheria treatment, its challenges in terms of clinical use and procurement, and current research to find alternative anti-diphtheria treatments.

Diphtheria agents and diphtheria toxin: recent taxonomic changes

Diphtheria, historically known as a «malignant tonsillitis», and in the public as the “strangling angel of children”, is an acute respiratory infectious disease caused by toxigenic strains of *Corynebacterium diphtheriae.* This bacterial species was discovered and cultivated by the German microbiologists Edwin Klebs (1883) and Friedrich Loeffler (1884). Toxigenic isolates generally carry the *tox* gene, which codes for the diphtheria toxin precursor, on a prophage, discovered back in the early 1950s, the first example of a phage-encoded virulence factor.^7^ These isolates produce DT, released into the culture medium as a 535 amino acid single chain protein, following cleavage of its 25 amino acid signal sequence. After invading the cell cytoplasm through endocytosis and translocation, DT inhibits protein synthesis and provokes cell death by apoptosis. DT is very potent towards humans, with an estimated lethal dose of 100 ng/kg.^8^

Colonial morphology variation among *C. diphtheriae* isolates was noted by Klein as early as in 1890, and was associated with biochemical variation and clinical severity by Anderson, McLeod and colleagues in the early 1930s.^9^ Subsequently, biochemical variation became central in defining three biovars of *C. diphtheriae*: Gravis, Mitis and Belfanti.^10^ Glycogen (or starch) fermentation and nitrate reduction characters can be used to distinguish these three biovars. A fourth biovar, Intermedius, was proposed by McLeod and colleagues^11^ as having biochemical characteristics of Mitis (lack of starch fermentation) but characteristics in culture medium that are intermediate between those that define Mitis and Gravis (barred bacillus forms, club shaped, no metachromatic granules). Tiny colonies on blood agar were proposed as a defining character.^12^ However, its existence as a distinctive variant was debated early,^9^ and this intermediate form is rarely reported in recent times, perhaps due to lack of recognition.^12^ Biovar Gravis was named with reference to the severity of infections and their partial refractoriness to eDAT treatment. In the 1940s, it was suggested that these strains produce more DT and hence cause more systemic and severe infections, due to a reduced inhibition of DT production in strains of this biovar, at the physiological concentrations of iron.^13^ However, there is a paucity of data on the links between biovar and infectious severity of recent clinical isolates.

The delineation of the taxon *C. diphtheriae* was refined recently, nearly a century later, by excluding most isolates of biovar Belfanti, which now belong to the two separate *tox*-negative species *C. belfantii* and *C. rouxii*.^14^ Nevertheless, strains of biovar Belfanti truly belonging to *C. diphtheriae* are observed occasionally, considered to evolve from Mitis strains by loss of the nitrate reduction character.^15^

Other species closely related to *C. diphtheriae* can carry the *tox* gene and can be toxigenic through its expression, *i.e*., can be shown to produce diphtheria toxin by using the Elek test*.* ^16^ ^,^ ^17^ Among these species, *C. ulcerans* is the most frequently reported in human infections in high-income countries.^18^ ^,^ ^19^ However, the exclusively zoonotic transmission of *C. ulcerans*, which is typically transmitted to humans through domestic dogs and cats, implies its exclusion as an agent of classical diphtheria, defined as the toxigenic *C. diphtheriae* respiratory infection transmitted among humans. Likewise, although *C. pseudotuberculosis* is sometimes considered as a potential agent of diphtheria, it causes a distinct disease spectrum (usually, granulomatous lymphadenitis), and is transmitted to humans by animals, mainly goats and sheep. Furthermore, *C. pseudotuberculosis* is an important pathogenic agent in multiple domesticated animal species including sheep, goats, horses and camelids,^20^ ^,^ ^21^ but only an exceptional subset of *C. pseudotuberculosis* isolates, isolated from water buffaloes in Egypt, was ever found to carry the *tox* gene. Differently, *C. ramonii* was defined recently from a subset of previously *C. ulcerans* isolates, and can also produce DT.^22^ Last, *C. silvaticum* is a zoonotic species that does not produce DT due to disruptions in its *tox* gene.^23^ For convenience, *C. diphtheriae* and the above-mentioned phylogenetically related species, some of which include toxigenic strains, are grouped into a taxonomic entity called the *C. diphtheriae* species complex.

As discussed above, DT-producing *C. ulcerans*, *C. ramonii* and *C. pseudotuberculosis* are not considered, under some strict definitions, to cause classical diphtheria, as they do not seem to transmit among humans (although evidence for zoonotic transmission is still lacking for *C. ramonii*). Still, they produce a very similar toxin, which is also neutralised by eDAT,^24^ and *C. ulcerans* can cause symptoms similar to those of classical diphtheria, with a severity that seems significantly reduced by vaccination against diphtheria.^18^ Clearly, the toxin produced by these other species is regarded as corresponding to DT,^24^ ^,^ ^25^ implying similar preventive and treatment guidelines. Diphtheria toxoid vaccination, which is prepared from detoxified DT of *C. diphtheriae* strain Park-Williams 8, raises antibodies that recognise DT from *C. ulcerans*.^26^ There is no randomised controlled trial data on efficacy of vaccination or eDAT treatment for *C. ulcerans*. Nevertheless, Wagner and colleagues suggested a protective effect of diphtheria vaccination on severity of *C*. *ulcerans* cases: none of 39 fully vaccinated cases presented with classic respiratory diphtheria with pseudomembrane, whereas 14 out of 43 unvaccinated or incompletely vaccinated cases presented with these symptoms (p < 0.001).^18^ In public health and clinical guidelines, diphtheria antitoxin treatment is recommended for toxigenic *C. ulcerans* upper airways infections in USA, UK and France.^27^ ^,^ ^28^ ^,^ ^29^

Diphtheria epidemiology and vaccination: from success to shortfalls

Before immunisation by diphtheria toxoid vaccination, which began in the 1920s, diphtheria was a highly contagious disease spread by infected individuals and was associated with large outbreaks. Hospital mortality was high, sometimes reported as high as 50%.^30^ In recent times, a meta-analysis estimated case fatality ratio for untreated, never-vaccinated cases at 29%, mostly (in 60% of cases) by asphyxia.^3^

Initially, immunisation was performed using a toxin-antitoxin mixture, and this approach began in the 1910s in America and Europe.^31^ In 1923 began the era of human toxoid vaccination, after Gaston Ramon demonstrated that formalin combined with heat eliminated the toxicity of the diphtheria toxin without altering its antigenicity or immunogenicity, thus introducing the anatoxin (generally called toxoid) principle.^32^ ^,^ ^33^ Moreover, in 1925, Ramon discovered adjuvants: “an inert substance which, when combined with an antigen, induces an immune response superior to that of the antigen administered alone”.^34^ In 1926, Glenny discovered aluminium adjuvants, used until today.^35^

Human diphtheria vaccination developed quickly, with a good efficacy in decreasing case numbers and diphtheria mortality.^36^ For example, in Ontario (Canada), diphtheria immunisation began slightly in 1916 (with toxin-antitoxin), and accelerated in 1925 with wide distribution of toxoid immunisation by Connaught laboratory. The diphtheria death incidence fell from 10/100,000 persons in 1923, to 1/100,000 in 1933.^37^ A comparison of toxin-antitoxin and toxoid strategies, including alum as an adjuvant, was published in 1932.^31^

In the 1940s, diphtheria toxoid, tetanus toxoid and pertussis antigens were combined in the diphtheria-tetanus-pertussis vaccine, still used widely throughout the world. World Health Organisation (WHO) Extended Programme on Immunisation vaccination program, launched in 1974, was very efficient in decreasing the number of annual diphtheria cases globally: from about 1 million cases in the 1970s before extended vaccination, to less than 60 000 after 1983 (Figure).^38^ ^,^ ^39^

**Figure:**
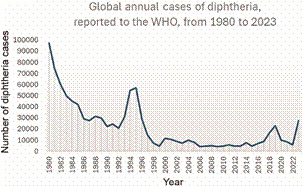
Global annual cases of diphtheria from 1980 to 2023, as reported to the World Health Organisation (WHO) (source: WHO, the global health observatory. Available from: https://www.who.int/data/gho/data/indicators/indicator-details/GHO/diphtheria---number-of-reported-cases).

Vaccine effectiveness is considered very high: about 94% after three doses and about 99% after five doses, although protection decreases with time.^38^ After the three-dose primary vaccination, three booster doses are needed during childhood and adolescence to ensure continuing protection throughout adulthood.^38^ A population vaccine coverage of 80-85% is considered associated with a reduced threat of epidemics in the corresponding population.^38^ A large meta-analysis of clinical studies of diphtheria estimated that the receipt of three doses of diphtheria toxoid vaccine was 87% effective against symptomatic disease and reduced transmission by 60%.^3^ Nevertheless, vaccinated individuals can become colonised and transmit; vaccination alone was estimated to interrupt transmission in only 28% of outbreak settings analysed in this meta-analysis, making isolation and antibiotics essential.^3^

Unfortunately, nowadays, some populations have no access to diphtheria vaccination, for example due to war or other causes of public health systems disruption. As a result, the number of reported diphtheria cases, which had been decreasing almost continuously since 1920, is now increasing again (Figure). Between 1997 and 2017, the number of reported cases of diphtheria was about 3,000 to 8,800 each year,^40^ whereas WHO reported 27,778 cases in 2023.^39^ In 2023-2024, large diphtheria outbreaks have been ongoing in West Africa (Nigeria, Guinea, Niger and other countries), with nearly 1,000 deaths reported as of 14 January 2024.^41^ Among the 18,500 diphtheria reported cases in these outbreaks, only 24% were fully vaccinated.^41^

In Europe, diphtheria cases have also been reported more frequently in the last years, with 224 cases reported in 2022, compared to an average of 55 cases reported annually between 2017 and 2021.^19^ Almost all of these cases are imported through travel or migration, typically in unvaccinated persons.^42^

Diphtheria toxin and associated clinical signs

Clinical presentations of infections by toxigenic members of the *C. diphtheriae* species complex include two main types of infections: respiratory and cutaneous. Symptoms range from the classical pseudomembranous pharyngitis and cervical lymphadenopathy to skin inflammatory ulceration; they develop with toxigenic or non-toxigenic strains. Toxin manifestations (myocardiopathy, neuritis, nephritis) are due exclusively to toxigenic strains.^43^ ^,^ ^44^ In addition, non-toxigenic strains can cause bacteraemia with or without endocarditis.^45^ The pathological effects of DT are described in more details below.

As early as in 1888, the two Pasteurian Emile Roux and Alexandre Yersin suspected the existence of the DT. In a seminal experiment, after removing the bacteria *C. diphtheriae* by filtration, they injected the bacterial culture broth subcutaneously to guinea pigs, and observed the same effects as with an injection of *C. diphtheriae* bacteria: oedema, kidney and adrenal haemorrhage, and death. The unique difference was the presence of cutaneous pseudomembrane in the *C. diphtheriae* injection group. Moreover, when they injected this diphtheria toxin to rabbits, paralysis was observed, similar to clinical signs in human diphtheria.^30^ These authors dubbed the potential factor, the “diphtheria poison”.

Today, we know that DT is an exotoxin secreted as a single polypeptide chain of 535 amino acid residues. DT enters host cells by binding to the membrane-anchored precursor of heparin-binding epidermal-growth-factor (hb-EGF), synthesised primarily by macrophages, but also by vascular endothelial and smooth muscle cells.^46^ ^,^ ^47^ In the cytosol, DT catalyses the ADP-ribosylation of elongation factor 2, an essential factor for protein synthesis, resulting in the inactivation of protein synthesis and leading to cell death by apoptosis.^48^ Interaction with the receptor is highly specific, contributing to the insensitivity to DT of some animal species, *e.g.*, rats and mice.^49^ In various animals, such as guinea pigs, monkeys, rabbits, fowl, DT can cause damage to multiple organs: heart, liver, kidneys, nervous tissue, supra-adrenal glands, pancreas, diaphragm.^50^

In humans, within one (1) to 12 weeks after the initial pharyngeal phase, DT may provoke many manifestations like cardiomyopathy (congestive heart failure, conduction abnormalities, arrhythmias), debilitating neurologic dysfunction (neuropathy of cranial or peripheral nerves, motor weakness, paralysis), or renal failure.^43^ ^,^ ^51^

*Cardiac effects of DT* - Toxic cardiomyopathy occurs usually seven to 14 days after the onset of respiratory symptoms in 10%-25% of patients and is responsible for 20%-25% of deaths.^3^ ^,^ ^43^ ^,^ ^52^ Patients present with an irregular pulse, evidence of heart failure with orthopnoea, peripheral and pulmonary oedema, hepatalgia and jugular turgescence.^51^ Conduction defects (auricle-ventricular block, branch block) and arrhythmias need specific cardiac treatment and can require, rarely, a temporary insertion of a cardiac pacemaker.^53^ Mortality is high without treatment: about 50% in one historical series in 1954;^43^ and around 8% nowadays.^52^

*Neurological effects of DT* - Neurological disorders, such as hypoesthesia, polyneuropathy, and cranial neuropathies, develop weeks to months after infection and occur in 15%-25% of untreated cases.^54^ ^,^ ^55^ They are responsible for up to 15% of deaths.^3^ Bulbar paralysis is the most common form and has been observed as early as the second day after the beginning of diphtheria illness.^54^ ^,^ ^55^ Peripheral paralysis is less frequent (about 1% of diphtheria cases), and occurs usually later, up to three months.^43^ ^,^ ^51^ Patients present with difficulty with swallowing (paralysis of the soft palate), vision (ocular motor paralysis), breathing (paralysis of respiratory muscles), hypoesthesia or limb paralysis. Ventilation dependent respiratory failure occurred in 20% of patients with neurological complications of diphtheria in Latvia in 1999.^54^ The mortality of bulbar paralysis was high in 1954: about 17%, probably partially due to one of its consequence: aspiration pneumonia.^43^ The patients described in Latvia in 1999 had no mortality due to neurologic complications (eight patients out of 50 died by cardiac severe cardiomyopathy or multiorgan failure). Nevertheless, they had neurologic sequelae one year later: 80% of limb symptoms, and 6% unable to walk independently.^54^

*Kidney effects of DT* - Kidney damage in diphtheria is less described than cardiac or neurologic ones. The kidney damage of DT was described as being frequent before the era of specific diphtheria treatments (antitoxin, vaccine and antibiotics). Since 1876, some severe patients have been described with diffuse oedema and albuminuria.^55^ ^,^ ^56^ In 1898, McCollom reported 800 cases of diphtheria and wrote “albuminuria is quite a constant symptom in diphtheria”.^57^ The same year, Councilman reports 103 autopsies on children who died from diphtheria, and found 24% of interstitial nephritis.^58^ In 2010, Turovets and Zriachkin report 47% of kidney lesions among 150 persons aged from 16 to 65 years in a pharynx diphtheria catamnesis.^59^ In 2024, Mahamadou and colleagues described 32 hospitalised patients with upper-airways diphtheria in Niger; 73% of them had altered renal function, with proteinuria (58%) and haematuria (42%).^60^ Most kidney damage presentations are interstitial nephritis^58^ ^,^ ^59^ and papillary necrosis.^59^ Interestingly, in a dog model of DT injection, albuminuria occurred quickly at 48 h, and the histological examination of kidneys showed tubular and glomerular lesions.^61^ Kidney damage is probably multifactorial. Nevertheless, taking into account the fast pathogenicity of DT in animal studies and since nephritic human cases are described only with toxigenic *C. diphtheriae*, DT is probably a major causative agent of kidney injury.

The discovery of DAT

Emil von Behring (1890) described experiments in which guinea pigs were immunised with old cultures of *C. diphtheriae* or with DT, and subsequently survived the lethal injection of virulent *C. diphtheriae* or DT.^1^ ^,^ ^2^ Moreover, this protection against DT toxicity was transferable to other animals. Von Behring also immunised rats with DT, and the serums of these rats were injected intraperitoneally to guinea pigs, who survived the lethal injection of DT. This “toxin destroying serum” was later named DAT, for diphtheria antitoxin.

In 1891, von Behring administered DAT to a seriously ill girl who subsequently recovered.^62^ This ushered the use of DAT in humans. To create large amounts of DAT, different animals were used, and finally horses were chosen. In 1891 in France, Edmond Nocard, from the Maison Alfort veterinary school, began the immunisation of horses to prepare large eDAT quantities. In Germany, horse immunisation for DAT began in 1892, with the contract between von Behring and the Germany-based company Hoechst. In 1893, Ehrlich joined the Hoechst company and standardised the strength of DAT, to optimise commercial production.^63^

Clinical trials with eDAT, and eDAT pharmacokinetics

In 1894, 220 children suffering from diphtheria and hospitalised in six institutions in Berlin, received cow DAT injections. The global survival was 76%, but there was no control group. The efficacy was greater when the DAT was administered early: 97% of children treated during the first two days of diphtheria symptoms remained alive, *versus* 55% when treated on the fifth day of the disease.^64^

In the same year, Roux and colleagues, from Paris Necker Children’s hospital, published the effect of two subcutaneous injections of eDAT on 300 children with confirmed diphtheria.^65^ The mortality was 26%, compared with 50% during the past years in the same hospital. Moreover, the mortality of children admitted in the diphtheria ward was lower than that of another hospital in Paris (Trousseau) at the same time: 24% (109/448) *versus* 60% (316/520).^65^

In Boston, eDAT use began in 1894. Subsequently, the diphtheria case fatality ratio in Boston fell from 31% between 1880 to 1894 to 13% in 1895-1897.^57^

However, the above studies were not randomised. The first controlled clinical trial, considered as randomised by some authors, was made by Fibiger in Copenhagen in 1896-1897.^66^ Patients were allocated to eDAT serum group or no serum group, according to the day of admittance, which created two arguably comparable groups. New patients admitted on alternate days received either standard treatment (silver nitrate or tar oil on the throat) or standard treatment plus subcutaneous injections of eDAT, twice daily until symptoms improved. More than 50% of admitted patients were excluded due to absence of identification of diphtheria bacteria, moribund status, measles, or scarlet fever. The study showed a significantly lower mortality in the eDAT serum group: 3% (8/239) *versus* 12% (30/245) in the control group; p = 0.0003. The efficacy of equine DAT serum *versus* no serum was arguably demonstrated by this early high-quality trial. The mortality in the control group was unexpectedly low, possibly due to exclusion of moribund patients in both groups. Investigators observed serum sickness (cf infra) very frequently: 60%, but without significant severity.^66^

After Fibiger’s study, to our knowledge, there was no randomised clinical trial comparing eDAT *versus* no serum. But, from 1913 to 1916, Bingel compared eDAT versus normal equine serum in Brunswick, Germany. This study is considered to have pioneered controlled double-blinded trials, with on average 460 diphtheria cases per group, with alternate allocation. The normal equine sera contained a very low concentration of anti-toxin (2 IU/cm3) compared to 500 IU/cm3 for eDAT serum. However, patients in the eDAT group received only 2,000 to 8,000 IU, a dosage much lower than recommended today. The mortality was the same in both groups: about 10%, leading to the hypothesis that horse serum itself, rather than specific anti-diphtheria antibodies, had the observed effect. Bingel himself repeated his trial a few decades later, with similar conclusions.^67^ ^,^ ^68^ These results may suggest that 2,000 to 8,000 IU of eDAT have no more observable effect than normal equine serum on diphtheria mortality. In 1942, the doses of eDAT used in the United States were higher: between 20,000 and 80,000.^43^ ^,^ ^69^.These eDAT doses are recommended by WHO, but with very low certainty evidence: the guideline development group wrote: “we are very uncertain which diphtheria antitoxin dosing regimen most effectively reduces mortality. However, the current standard of care ― escalating dosing regimens ― is well established in clinical practice globally.^69^

A recent meta-analysis found that eDAT reduces mortality by 76%.^3^ However, eDAT is effective only when it is administered early, *i.e.*, within two-three days after the beginning of diphtheria signs.^3^

What is the pharmacokinetics of eDAT? Smith and colleagues estimated the human serum concentrations of antitoxin antibodies after eDAT injection, from four patients that were treated with eDAT for suspected diphtheria in the USA.^70^ None of the patients had diphtheria infection confirmed by culture or polymerase chain reaction (PCR). Three subjects received a single dose of 80,000 IU eDAT intravenously (as recommended by the CDC diphtheria duty officer in 2017), and one patient received 100,000 IU. Blood samples were collected 1 h, one (1) day, three days and after seven days post injection, and led to observe a mean of the maximum concentration of equine anti-diphtheria binding antibodies of 19 U/mL (by ELISA). Moreover, their sera were used to determine anti-diphtheria toxin neutralising activity, by Vero cell cytotoxic assay. Maximum serum neutralising activity ranged from 28 to 39 Antitoxin Units/mL. A mean half-life of 78 h was inferred by using binding and neutralising data. Despite the small number of subjects, these data contribute to provide a standard of comparison for development of novel anti-toxins in order to replace eDAT,^70^ *e.g.*, it could represent a target concentration in future phase 1 trials of a new anti-toxin treatment.

Current eDAT treatment guidelines for diphtheria

Today, the recommended treatment for diphtheria is antibiotic therapy, combined with eDAT in case or risk of toxinic signs.^69^ We describe antimicrobial therapy further below.

eDAT is indicated for probable or confirmed cases of respiratory toxinic diphtheria in the majority of national guidelines, and in WHO guidelines.^28^ ^,^ ^29^ ^,^ ^51^ ^,^ ^69^ In France, it is indicated in addition for toxigenic cutaneous diphtheria with toxigenic signs.^29^ eDAT is preferably administered intravenously, or intramuscularly for moderate cases, and the total dose is given in one injection, added to the optional test dose. The posology is the same for adults and children. It depends on the type of clinical presentation of diphtheria in most national guidelines. For example, in WHO current guidelines, cutaneous or pharyngeal/laryngeal cases of two days duration require 20,000 eDAT IU; nasopharyngeal diseases less than 48 h duration require 40,000 IU. A dose of 80,000 eDAT IU is recommended for any disease of more than 48 h duration, or diffuse swelling of the neck or severe disease (respiratory distress, shock).^69^ Only one dose is recommended, as repeat dosing can cause hypersensitivity.

To avoid immediate hypersensitivity reaction (anaphylaxis), an injection of a lower dose (“test dose”) is sometimes recommended, intravenously or subcutaneously, with details determined by each manufacturer. However, for the 709 patients treated with eDAT during the 2017/2018 Bangladesh diphtheria outbreak, eDAT sensitivity testing was stopped after the first 2 weeks because clinicians noted that skin-test results were poorly predictive of which patients got adverse reactions.^71^ These patients, premedicated with steroids and antihistamines 30 min prior to eDAT infusion, had few severe drug reactions (described below). In 2024, WHO diphtheria guidelines mention that test dose and premedication are not necessary.^69^ WHO recommends to ensure sufficient trained staff and equipment are available: monitoring, emergency equipment and emergency medicines (adrenaline, bronchodilator, antihistamines, corticosteroids, high flow oxygen).

The role of antimicrobial therapy

Antibiotics should be administered alongside eDAT, and should not be delayed. Penicillin (penicillin G intravenous or intramuscular; penicillin V oral) or macrolides (azithromycin, erythromycin) have been recommended as first line antimicrobial therapies.^51^ In France, amoxicillin is recommended, and macrolides in case of allergy to beta-lactams.^72^ Recent guidelines recommend a macrolide, in combination with penicillin in severe cases. Indeed, in UK 2023 guidelines, for mild disease, such as small cutaneous lesions with no evidence of systemic toxicity, the preferred empirical antibiotic is a macrolide (either clarithromycin, azithromycin or erythromycin). For severe disease, intravenous benzylpenicillin should be combined with a macrolide; in patients who are both extremely and systemically unwell, a third agent such as IV vancomycin or linezolid is considered until local susceptibility results are available.^28^

In February 2024, in the context of the 2023 West African diphtheria outbreak, WHO published a new guideline for the clinical management of diphtheria, where the first-line use of macrolide antibiotics (azithromycin, erythromycin) is recommended in preference to penicillin.^69^ This strong recommendation is acknowledged to be of low certainty evidence by the WHO guideline development group; it is based on the evaluation of benefits and harms of macrolides and penicillins for diphtheria patients, notably one randomised trial comparing penicillin *versus* macrolides.^73^ The guideline adds that in the circumstances where antitoxin is unavailable and unlikely to be accessible in a short period, there is a speculative benefit of dual antibiotic treatment. In such cases, where bacteriological susceptibility is unknown, clinicians might choose, pending susceptibility data, to treat concurrently with both macrolide and beta-lactam antibiotics. Anyway, as with other bacterial infections, antimicrobial use should be adapted during treatment based on antibiograms.

Antimicrobial resistance is reported in *C. diphtheriae* for a number of agents, including penicillin and erythromycin, but is highly variable across studies, time and geographic areas.^15^ ^,^ ^60^ ^,^ ^73^ ^,^ ^74^ ^,^ ^75^ Penicillin resistance reporting may in addition depend on interpretation guidelines, as critical breakpoints are not consensual.^76^ ^,^ ^77^ Ideally, antimicrobial susceptibility testing should be performed through continuous local surveillance and during outbreaks, in order to guide the appropriate use of antibiotics. Methodology and interpretation guidelines for *C. diphtheriae* and *C. ulcerans* antimicrobial susceptibility testing were recently proposed by the European Committee on Antimicrobial Susceptibility Testing (EUCAST).^77^

Antibiotics play a key role in reducing infectiousness and transmission.^3^ Indeed, patients receiving antibiotic treatment eliminate *C. diphtheriae* respiratory colonisation within 5.2 days of initiating treatment on average, thus reducing the average duration of infectiousness by as much as two weeks.^3^

Adverse reactions to eDAT

Adverse reactions to eDAT include anaphylaxis, early fever, serum sickness, aspecific mild cough and cutaneous reactions. They are mostly due to horse serum hypersensitivity, immediate or delayed.

Adverse reactions of eDAT were very common before 1900, *e.g.*, 60% in Fibiger’s study.^66^ They became less frequent with purification of eDAT. For example, 8.8% of 1433 patients had adverse effects in the retrospective study of Naiditsch (1954).^43^ In that study, adverse effects were divided into three types: immediate reactions during the test injection or the first full injection (0.6%; all 8 patients recovered); fever in the first 24 h (4.1%) and serum sickness between the second and the eighteenth day (4.1%). In 2017, WHO mentions the frequencies of 0.6% for immediate anaphylaxis, 4% for early fever (within 20 to 60 min) and 8.8% for serum sickness (between five and 25 days).^51^ Recently, Eisenberg^71^ described the side effects of 709 patients who received eDAT during the diphtheria outbreak in Cox’s Bazar in Bangladesh (2017-2018). An Indian F(ab)2 enzyme- refined eDAT was used, obtained after pepsin digestion, heat inactivation and caprylic acid precipitation of hyper-immune plasma of healthy horses. Despite premedication with steroids and antihistamine 30 min prior to eDAT intravenous infusion, the authors observed 24% probable adverse effects: 3% immediate anaphylaxis, 16% cough, 9% cutaneous benign reactions and 5% itching.^71^ Cough had no clear origin: it could be due to diphtheria itself rather than to drug hypersensitivity. These side effects were not life-threatening. The overall mortality was 1%, none being attributed to adverse effects. Another recent study^78^ supported Eisenberg and colleagues’s conclusions on the safety of eDAT: among a smaller sample size (18 patients in the United States) and a different eDAT product, the authors observed few adverse events (6%), which were mild (fever and maculopapular rash). Nevertheless, since there are different eDAT manufacturers, adverse reactions may vary from one product to another.

Current global eDAT shortage

In 2016, the WHO strategic advisory group of experts expressed concern over the lack of eDAT.^79^ eDAT supply availability from manufacturers has been limited for many years.^80^ The number of manufacturers has decreased from nine in 2016 to six in 2019.^29^ The current eDAT active suppliers are, to our knowledge, Bulbio (Bulgaria), Instituto Butantan (Brazil), Premium Serums (India), Haffkine Biopharmaceuticals (India), biological E (India), Vins Bioproducts (India) (WHO supplier survey to be released).

In the European Union, based on a gap analysis published in 2017, only half of countries reported that they had an existing stockpile.^81^ This is the case in France, and antitoxin is delivered with a “compassional access authorisation”.^29^ During large outbreaks, there is tension on eDAT procurement; this was the case during the 2023-2024 West African diphtheria outbreak.^82^

There are currently no other forms of DAT than the equine’s one.

The prospects of alternative treatments to eDAT

Today, no DAT treatment other than the classical eDAT one is approved for use, and none is even in phase II or III clinical trial evaluation. This lack of pharmaceutical development reflects, in part, the success of vaccination. Nevertheless, with the resurgence of diphtheria and experienced difficulties of eDAT procurement, interest in novel diphtheria therapeutics is growing again.

The preclinical development path is defined as follows: after producing antitoxin products, their efficacy can be tested in an *in vitro* Vero cell cytotoxicity protection assay, and then, in an *in vivo* guinea pig lethality model.^83^

A single-target therapeutic strategy aimed at neutralising DT represents a well-defined objective. Another advantage is that DT is highly conserved among bacterial strains, including in *C. diphtheriae* and *C. ulcerans*,^25^ which increases the likelihood that a neutralising antibody, or set of antibodies, will be a successful “broad-spectrum” therapeutic against diphtheria.

Human antibodies have the advantage of being safer than equine ones, because hypersensitivity reactions to human antibodies are much less common. Human polyclonal antibodies from plasma from young adults receiving a booster dose of diphtheria toxoid-containing vaccine could represent a potential source of human DAT, but evaluations suggest a need for a large volume of plasma (about 1.6 litres) to achieve 100,000 IU DAT.^84^ To produce a highly concentrated DAT from this plasma, antigen-affinity antibody purification could be a solution. Nevertheless, this approach may be very costly and needs large quantities of plasma, which is difficult to procure nowadays for many medical indications.^84^

Human monoclonal recombinant antibodies (hMAb) may represent another option. In 2013, Sevigny and colleagues obtained a potent human DAT monoclonal antibody, directly from antibody-secreting cells, using the plasma of three volunteers who had recently received a diphtheria vaccination. This G immunoglobulin (IgG) hMAb, named 315C4, was effective in *in vitro* cytotoxicity assays with a 50% effective concentration of 0.65 ng/mL. In addition, 25 micrograms of 315C4 completely protected guinea pigs from intoxication in an *in vivo* lethality model, yielding an estimated relative potency of 64 IU/mg (in comparison, 1.6 IU of eDAT was necessary for full protection from morbidity and mortality in this model). The authors further established that this hMab binds to the receptor binding domain of DT, and thereby blocks the interaction with its putative receptor, hb-EGF.^83^ Subsequently, this hMAb (named S315) was tested in a guinea pig model with more animals.^85^ The authors injected the toxin mixed with the human monoclonal antibody at different dosages, and they monitored impact on observed mortality and signs of neuropathy. The same was done with eDAT. The results showed a good efficacy of S315 and allowed determining S315’s potency, by comparison with eDAT. The potency estimate was 17 µg S315/IU DAT (95% CI 16-21) for an endpoint of survival and 48 µg S315/IU DAT to prevent limb paralysis. In 2020, a phase I clinical trial was completed, evaluating safety and pharmacokinetics of S315 in healthy individuals (Trial ID NCT04075175). The results should be available soon.

Following a distinct route, Wenzel and colleagues generated different human recombinant antibodies against DT, from the plasma of a volunteer who had recently received a diphtheria vaccination.^86^ In this work, 400 human recombinant antibodies were generated against DT from two different phage display panning strategies. Sixty-one unique antibodies were further characterised as single-chain variable fragment-constant fragment (scFv-Fc) with 35 produced as fully human IgG1. The best *in vitro* neutralising antibody showed an estimated relative potency of 454 IU/mg. Using an *in vivo* intradermal challenge assay in guinea pigs, the authors demonstrated that a combination of two to three recombinant antibodies was associated with a better DT neutralising capacity than an individual recombinant antibody.^86^

These hMAb represent promising candidates as novel DAT therapies. The advantages include a sequence-defined human antibody produced under controlled and reproducible conditions in cell culture, as opposed to an animal blood product with batch-to-batch variation and risk of adverse reactions.

Conclusions: how long will diphtheria antitoxin treatment remain a technology of the 19th century?

Diphtheria cases are rising worldwide, and are now observed in numbers unseen for two decades. Antitoxin therapy based on eDAT, a treatment invented in 1890, is still the only available treatment to specifically target the toxic effects of DT. Today, eDAT procurement is a bottleneck during the public health response to diphtheria epidemics, as experienced during the West Africa 2023-2024 outbreak. More than 130 years after its invention, there is no alternative treatment despite the biotechnology capacity of the 21st century. Novel antibody-based therapeutic strategies have proven effective for other pathogens, with high efficacy and safety. For example, human antibodies derived from plasma of human donors are used routinely for the post-exposition treatment of rabies, hepatitis B, tetanus and measles. Monoclonal recombinant antibodies have extended indications in infectious diseases, *e.g.*, against severe acute respiratory syndrome coronavirus 2 (SARS-CoV-2),^87^ Ebola,^88^ human immunodeficiency virus (HIV),^89^ ^,^ ^90^ with an efficacy that is optimised by using multiple monoclonal antibodies simultaneously. Promising results from studies of recombinant human antibodies directed against DT indicate hope. However, these treatments are desperately needed and clinical trials are very long to perform, and costly. There is an urgent need to promote these developments in order to accelerate the availability of novel DAT strategies for diphtheria patients.

Although vaccination is highly effective, its suboptimal implementation when public health systems are disrupted means it is not going to prevent all diphtheria outbreaks, as illustrated in the last years, and these continue to have high fatality rates.^60^ Antimicrobial treatment reduces transmission, but to improve survival, patients with toxinic symptoms do need DAT. The resurgence of diphtheria cases, associated with poor vaccination coverage in multiple settings and shortage of existing eDAT products, underlines the urgent need for alternative DAT strategies.
